# Head Computed Tomographic measurement as an early predictor of outcome in hypoxic-ischemic brain damage patients treated with hypothermia therapy

**DOI:** 10.1186/1757-7241-21-37

**Published:** 2013-05-14

**Authors:** Hitoshi Yamamura, Shinichiro Kaga, Kazuhisa Kaneda, Tomonori Yamamoto, Yasumitsu Mizobata

**Affiliations:** 1 Department of Critical Care Medicine, Osaka City University Graduate School of Medicine, Osaka, Japan

**Keywords:** Hypoxic-ischemic brain damage, Neurological outcome, Post-cardiac arrest syndrome

## Abstract

**Background:**

Neurological abnormalities are a key factor in the prognosis of patients with post-cardiac arrest syndrome. In this study, we evaluated whether differences in CT measurements expressed in Hounsfield units (HUs) of the cerebral cortex and white matter can be used as early predictors of neurological outcome in patients treated with hypothermia therapy after hypoxic-ischemic brain damage.

**Methods:**

We performed a retrospective study of 58 patients resuscitated after cardiac arrest between 2007 and 2010 who were treated with hypothermia therapy for the initial 24 hours post resuscitation. We divided the patients into 4 groups according to Glasgow Outcome Scale (GOS) score (GOS 1, GOS 2, GOS 3&4, and GOS 5) and assessed the correlations between GOS scores and HU differences between the cerebral cortex and white matter (DCW).

**Results:**

The HU values of the cerebral cortex gradually decreased in accordance with worsening of neurological outcome. There were no significant intergroup differences in the HUs of the white matter among the groups. The DCW values were higher in patients with good neurological outcomes. The cut-off value for DCW indicative of poor neurological outcome was less than 5.5 in the GOS 1&2 groups, with a sensitivity of 63% and a specificity of 100%.

**Conclusions:**

This study showed that DCW values may be used for the prediction of neurological outcome of patients with post-cardiac arrest syndrome in the very early phase following the return of spontaneous circulation. Especially, a cut-off value for DCW of less than 5.5 may indicate poor neurological outcome.

## Introduction

Post-cardiac arrest syndrome consists of several symptoms that resuscitated patients exhibit after cardiac arrest, such as neurological dysfunction, cardiac failure, and respiratory distress [1]. Of these symptoms, neurological dysfunction is a key factor for determining the prognosis of patients post cardiac arrest. Hypothermia is a helpful therapeutic approach for the protection of brain cells in patients who remain comatose after the return of spontaneous circulation (ROSC). Preclinical and clinical evidence strongly supports therapeutic hypothermia as an effective therapy for post-cardiac arrest syndrome [1].

Head computed tomography (CT) is often performed early following ROSC to evaluate brain hemorrhage or infarction in post-cardiac arrest patients. From our experience with head CT findings in such patients, we found that patients with hypoxic-ischemic brain damage following cardiac arrest showed a loss of difference between gray matter (GM) and white matter (WM), diffuse brain swelling, and decreased basal ganglia density [2,3]. Several studies have examined the correlations between CT findings and the prognosis of patients with post-cardiac arrest syndrome. Torbey et al. demonstrated a correlation between GM/WM ratios measured at the level of the basal ganglia and neurological outcomes in patients after ROSC [4]. A loss of GM/WM differentiation on CT has been reported to predict poor outcome after hypoxic brain damage and hypoxic encephalopathy. However, no studies have evaluated CT scans that were performed within a few hours of ROSC and the outcome of patients after ROSC.

This study aimed to evaluate whether differences in CT measurements expressed in Hounsfield units (HUs) of the cerebral cortex and WM can be used as early predictors of neurological outcome in patients undergoing therapeutic hypothermia after hypoxic-ischemic brain damage.

## Methods

Of 310 adult patients who had been resuscitated after cardiac arrest in our center between October 2007 and January 2010, we retrospectively evaluated 58 patients who had undergone head CT within 2 hours of ROSC and survived 24 hours after ROSC and who did not meet the following exclusion criteria: 1) intracranial hemorrhage or brain infarction as a cause of cardiac arrest, and 2) death within 48 hours from respiratory or heart failure. Spontaneous circulation did not return in 183 of the 310 patients resuscitated. The remaining 69 patients were excluded for the following reasons: intracranial hemorrhage or brain infarction (n = 29); lack of CT data (n = 18); and death within 48 hours, which included patients whose families did not desire hypothermia treatment (n = 22).

The patients in this study were treated with constant hypothermia therapy for its curative effect. They were cooled to a temperature range of 33°C to 35°C for the initial 24 hours after ROSC with external cooling devices, which included water-circulating cooling blankets (Medi-Therm® III; Gaymar Industries, Inc., New York, NY, USA) or the Arctic Sun® 5000 system (Medivance, Inc., Louisville, CO, USA). All patients were given sedatives and neuromuscular blockade to prevent shivering.

All data pertaining to clinical presentation, including age, sex, cause of death, witnessed collapse, bystander cardiopulmonary resuscitation, resuscitation time, time between initial CT scan and ROSC, and Glasgow Outcome Scale (GOS) scores, were collected from patient medical records. Neurological outcome of the patients was assessed with the GOS score at the time of discharge from hospital.

We divided the patients into 4 groups according to GOS score: 1 (GOS 1 group, n = 24), 2 (GOS 2 group, n = 18), 3 and 4 (GOS 3&4 group, n = 6), and 5 (GOS 5 group, n = 10). The head CT scans of 20 pre-cardiovascular surgery patients, which were interpreted by radiologists to be within normal range, were used as the control group.

**Figure 1:**
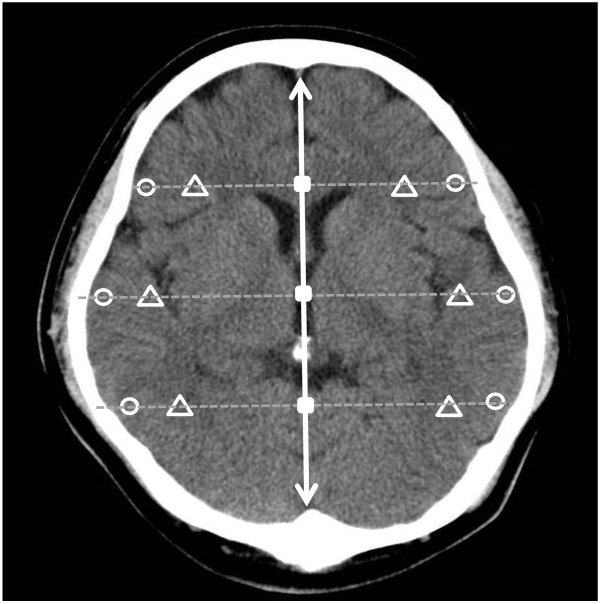
**Measurement points of head computed tomography at the basal ganglial level.** The indicated points were measured in Hounsfield units. Open circles indicate the positioning of the 10-mm^2^ regions of interest in the cerebral cortex. Open triangles indicate the positioning of the regions of interest in the white matter 1. Square symbols indicate the hallmarks placed to divide the distance from the frontal pole to occipital pole into four equal parts.

CT scan images were obtained on an Aquilion™ CT system (Toshiba, Tokyo, Japan). All CT images were non-contrast-enhanced 5-mm axial sections obtained parallel to the orbitomeatal baseline at 135 kV and 150 mA. Images were viewed on a 512 × 512-pixel monitor on an electrical communication system. We obtained the HU measurements of the cerebral cortex and WM1 at 6 points (bilateral frontal, temporal, and occipital lobes). A radiologist standardized the placement of measurements at the basal ganglia level. We drew a center line from the frontal pole to the occipital pole and placed hallmarks at the quarter, halfway, and three-quarter points to divide the length into 4 equal parts. We then drew lines perpendicular to the center line to the right and left of these three hallmarks and placed ROIs (10 mm^2^) along these lines at 2 mm inside the cranial bone on either side as measurement points of the “cerebral cortex” (denoted by circle symbols) and at 15 mm inside of these points as measurement points of the “WM1” (denoted by triangle symbols) (Figure 1). We also obtained the HU values of the GM and WM using the method described by Torbey et al., whereby GM values were obtained from the caudate nucleus and putamen and WM2 values were obtained from the posterior limb of the internal capsule and genu of the corpus callosum at the basal ganglia level on non-contrast CT scans [4]. We selected these points to avoid beam-hardening artifacts of bone. The average values of the cerebral cortex and of the WM1 at the 6 points were recorded as the respective values for the cerebral cortex and WM1 in that area.

The difference in HU values between the cerebral cortex and WM1 (DCW) was calculated as the difference between the mean HU value of the cerebral cortex and mean HU value of the WM1. We also assessed the correlations between the GOS scores and the DCW. These assessments were performed after patients were discharged from hospital, and all CT scans were evaluated independently on an X-ray viewer in the intensive care unit by an emergency physician who was blinded to the clinical findings and patient data. The study was approved by the local ethics committee of our university hospital.

### Statistical Analysis

Data are expressed as mean ± standard deviation (SD). Differences in HUs between the cerebral cortex and WM1 between the controls and the 4 subgroups were compared with one-way analysis of variance (ANOVA) and post hoc comparison using Scheffé’s method. Using the ROC method, we calculated the sensitivity, specificity, and area under the curve (AUC) to determine the diagnostic accuracy of our findings. *P* values of less than 0.05 were considered significant.

## Results

Patient characteristics are presented in Table 1. There were significant differences between the 4 GOS groups with respect to witnessed arrest, time interval between cardiac arrest and ROSC, and time interval between ROSC and CT. The mean time intervals between cardiac arrest and ROSC were shorter in patients with good neurological outcomes. The mean duration from ROSC to attainment of the hypothermia target temperature (35°C) was 115 + 104 min. The temperature of 16 patients at ROSC was below 35°C. The number of hospital days was 21 ± 21 days (range, 3 – 96 days). In the patients in GOS 1 group who died, duration of survival was 4.6 ± 2.1 days (range, 3 – 9 days).

**Table 1:** Clinical characteristics of the 4 patient groups

	**Glasgow outcome scale**				
	***1***	***2***	***3&4***	***5***	***P***
No.	24	18	6	10	
Male/Female, N	17/7	15/3	5/1	5/5	0.442
Age, years	65 ± 16	66 ± 18	73 ± 15	65 ± 11	0.754
Time between cardiac arrest and ROSC, min	53 ± 22	38 ± 12	30 ± 4	20 ± 15	<0.0001*
Time between ROSC and CT, min	35 ± 16	35 ± 11	60 ± 45	65 ± 50	0.012*
Witnessed arrest,%	24	61	83	90	<0.001*
Cause of cardiac arrest					
Cardiogenic	2	10	4	8	
Asphyxia	14	3	1	0	
Others	8	5	1	2	

ROSC, return of spontaneous circulation; CT, computed tomography.

* Statistically significant (*P* < 0.05).

The HU densities of the cerebral cortex, caudate nucleus, and posterior limb of the internal corpus callosum, and the DCW were significantly lower in the GOS 1&2 groups than in the GOS 3–5 groups (Table 2). The GM/WM2 ratios were not significantly different between these two composite groups.

**Table 2:** Assessment of HU values in regions of interest and the gray matter/white matter 2 ratio in GOS 1&2 and 3–5 patients

	**GOS 1&2(n = 42)**	**GOS 3–5(n = 16)**	***P***
***Measurement by our original method***			
**Cerebral cortex**	32.1 (2.9)	36.2 (1.4)	<0.001
**White matter 1**	29.8 (2.3)	30.1 (1.2)	0.69
**DCW**	2.2 (1.6)	6.1 (1.4)	<0.001
***Measurement by Torbey et al. method***			
**Gray matter**			
Caudate nucleus	34.6 (2.5)	36.7 (1.4)	0.006
Putamen	33.4 (2.5)	35.3 (2.4)	0.21
**White matter 2**			
Posterior limb of internal capsule	30.9 (2.6)	32.4 (1.7)	0.04
Corpus callosum	29.5 (2.5)	30.4 (1.7)	0.21
**GM/WM2 ratio**	1.12 (0.1)	1.13 (0.1)	0.71

Values are mean (SD). HU, Hounsfield unit; GOS, Glasgow Outcome Scale; SD, standard deviation; DCW, difference in HU values between the cerebral cortex and white matter 1; GM, Gray matter; WM2, White matter 2.

**Figure 2:**
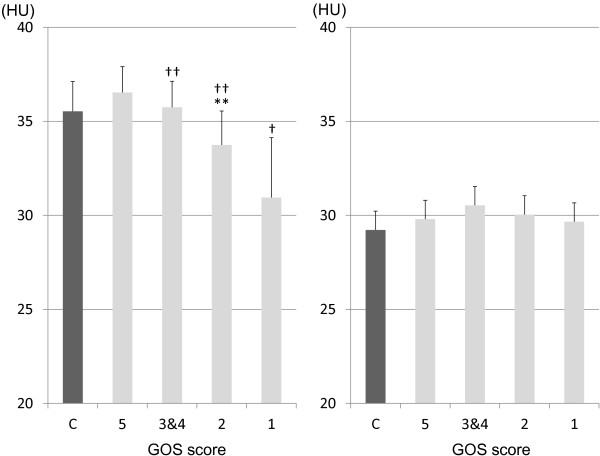
**Hounsfield units (HU) of the cerebral cortex (left) and white matter 1 (right) of 4 the groups.** There were no significant intergroup differences in the HU values of the white matter 1 among these groups. C, Control. †*P* < 0.001 vs C and GOS 5 groups, **P < 0.01 vs C and GOS 5 groups, ††*P* < 0.001 vs GOS 1 group.

The HUs of the cerebral cortex and WM1 are shown in Figure 2. The HU values of the cerebral cortex gradually decreased in accordance with worsening of neurological outcome. There were no significant intergroup differences in the HUs of the WM1 between the groups.

**Figure 3:**
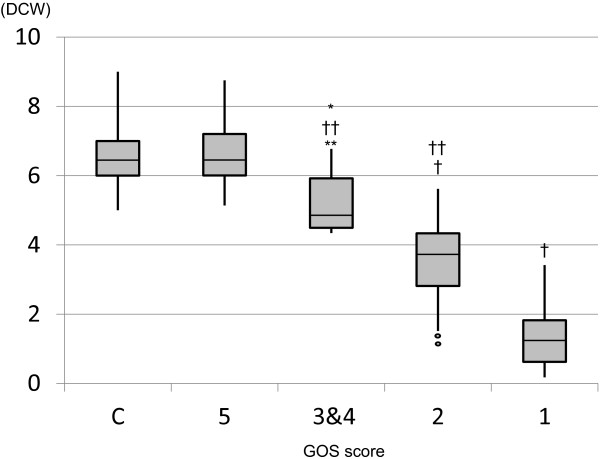
**Differences between the cerebral cortex and white matter 1 (DCW) values among the groups.** The DCW values on the Y axis gradually decreased in accordance with worsening of neurological outcome. Box and whisker plots with median, lower and upper quartiles, and outliers are shown. C, Control. †*P* < 0.001 vs C and GOS 5 groups, **P < 0.01 vs C and GOS 5 groups, ††*P* < 0.001 vs GOS 1 group, *P < 0.001 vs GOS 2 group.

The DCW values in the control and GOS 5 groups were significantly higher than those in the GOS 3&4 (*P* < 0.01), GOS 2 (*P* < 0.001), and GOS 1 (*P* < 0.001) groups (Figure 3). The DCW value in the GOS 3&4 group was significantly higher than that in the GOS 2 group (*P* < 0.001), and the DCW value in the GOS 2 group was significantly higher than that in the GOS 1 group (*P* < 0.001). In addition, the DCW values in the GOS 3,4,5 groups were significantly higher than those in the GOS 1&2 groups (*P* < 0.001).

Using ROC analysis, we determined that a DCW cut-off value of less than 5.5 was predictive of poor neurological outcome defined as GOS 1&2, with a sensitivity of 63%, specificity of 100%, positive predictive value of 100%, negative predictive value of 86%, and AUC 0.93.

## Discussion

A neurological predictor of mortality from post-cardiac arrest syndrome is important from the perspectives of containing costs and explaining to patients’ families issues concerning neurological outcome. Predictors such as neurological examinations, electroencephalograms, and biochemical markers, which are influenced by sedative drugs or hypothermia therapy, are difficult to apply [5,6]. Most previous studies have collated CT findings and neurological outcomes after ROSC in patients who were not treated with hypothermia therapy [4,7-9].

ROSC after prolonged, complete, whole-body ischemia is an unnatural pathophysiological state that is created by successful cardiopulmonary resuscitation. Recently, post-cardiac arrest syndrome has been defined to consist of a complex combination of pathophysiological processes that include hypoxic brain damage, post-cardiac arrest myocardial dysfunction, and systemic ischemia/reperfusion response [1,10,11]. Post-cardiac arrest syndrome is potentially treatable, although survival and neurological outcome depend essentially on time factors following the arrest. Post-cardiac arrest care has significant potential to reduce early mortality caused by hemodynamic instability and later morbidity and mortality from multiple organ failure and brain injury. The objectives of post-cardiac arrest care are to optimize ventilation and oxygenation, treat hypotension and acute coronary syndrome, apply hypothermia therapy, avoid mechanical lung injury, and reduce multiple organ failure [1,12].

Hypothermia is a helpful therapeutic approach for the protection of brain cells in patients who remain comatose after ROSC [13-15]. Two randomized clinical trials and a meta-analysis have shown improved outcomes in adults who remained comatose after initial resuscitation from out-of-hospital ventricular fibrillation cardiac arrest and who were cooled within minutes to hours after ROSC [16-18]. The 2010 American Heart Association Guidelines for Cardiopulmonary Resuscitation and Emergency Cardiovascular Care recommend that comatose adult patients with ROSC after out-of-hospital ventricular fibrillation, pulseless electrical activity, or asystole cardiac arrest should undergo hypothermia cooling for 12 to 24 hours [12].

Our finding that decreasing DCW values were obtained on head CT scans within a very early time from ROSC was surprising. HU values of the cerebral cortex gradually decreased in accordance with worsening of neurological outcome in just a few hours after ROSC. However, the HU values of the cortical WM1 did not change relative to neurological outcome. HU changes depend on the water content in brain tissue, and there is more water content in the cerebral cortex than in the WM [19]. If the ischemic insult that occurs to the brain results in edema, the edema occurs more often in the cerebral cortex than in the WM.

Several reports have discussed head CT findings in patients with anoxic brain damage. Torbey et al. showed a correlation between GM/WM ratios measured at the level of the basal ganglia and neurological outcomes in patients after ROSC [4]. A loss of GM/WM differentiation on CT has been reported to predict poor outcome after hypoxic brain damage and hypoxic encephalopathy. In addition, Choi et al. reported that the density ratio of GM to WM on head CT images that were obtained within 24 hours of ROSC correlates with outcome in comatose patients after cardiac arrest [7]. However, the patients in both of these studies had undergone head CT within 24 hours of ROSC.

In our observation of the density ratio of GM to WM2 on head CT images early after ROSC by Torbey’s method, we found no significant difference in the GM/WM2 ratio between the GOS 1&2 and GOS 3–5 composite groups. We thought the reason for this result was that HUs of the cerebral cortex were more changeable than HUs of the posterior limb of the internal capsule or caudate nucleus in the very early time after ROSC due to differences in the water content of each tissue and in how hypoxia exerts its influence in a tissue-dependent manner.

We determined the measurement of DCW in our study based on the following reasoning. First, we realized that in cases of hypoxic-ischemic brain damage following cardiac arrest, CT findings show a loss of difference between GM and WM in the presence of diffuse brain swelling. We intended to measure the HU differences between the cerebral cortex and WM1 on the basis of findings showing a loss of difference between the GM and WM. Second, we measured GM and WM2 using the method described by Torbey et al. but found that in the very early phase after ROSC, the GM/WM2 ratio did not correlate with neurological prognosis.

Our findings suggested that DCW may be one predictor of neurological outcome of patients in the early phase after cardiac arrest. The most important finding of the present study was that prediction of neurological outcome could be made within a few hours after ROSC.

With respect to patient background, there were some differences in each group. In the GOS 5 group, the percentage of witnessed arrest patients was 90%, but that in the GOS 1 and 2 groups was 29% and 61%, respectively. The time interval between cardiac arrest and ROSC was longer in patients without good neurological outcomes. These results suggested that the duration of hypoxia in the GOS 1 and 2 groups was longer than that of the GOS 5 group.

The cut-off value for DCW predicting poor neurological outcome was less than 5.5 in the GOS 1&2 groups, with a sensitivity of 63% and specificity of 100%. The usefulness of the DCW cut-off value as a prognostic indicator in the present study was comparable to that of the previous method using the cut-off value for GM/WM ratio of less than 1.22 [7], which predicted a poor neurological outcome with lower sensitivity and specificity.

Recently, the medical staff at our hospital has been able to view X-ray and CT films of electronic charts at various computer terminals throughout the hospital. This system allows easier access to measured HU values and distance and angle measurements of images in the intensive care unit or in other wards. We are now able to quickly and easily assess HU values of patients with post-cardiac arrest syndrome throughout our hospital.

Our study has several limitations. First, this was a retrospective, uncontrolled study. However, all brain CT scans were performed according to a standardized protocol. Second, HU values are affected by beam hardening artifacts of bone or air. Therefore, we very carefully selected the measurement points on CT images to avoid this. Third, although the groups seemed similar in all baseline comparisons (Table 1), there were differences between the groups in sex ratio, percentage of witnessed arrests, and time interval between cardiac arrest and ROSC. Fourth, the small sample size of the study group could have obscured differences in some of these parameters. Therefore, a well-designed prospective study of a larger number of patients is needed to confirm the cut-off value for DCW that will predict neurological outcome for patients with post-cardiac arrest syndrome.

## Conclusions

Our study showed that DCW values obtained from CT scanning performed within a very short time after ROSC may be used for the prediction of neurological outcome of patients with post-cardiac arrest syndrome. Especially, a cut-off value for DCW of less than 5.5 may be predictive of poor neurological outcome.

## Competing interest

The authors declare that they have no competing interests.

## Authors’ contributions

HY and YM contributed to the conception and design of this study. HY, SK, and KK evaluated patient eligibility for the study. HY and YM carried out and reviewed the analysis. HY and TY drafted the manuscript. All authors participated in interpretation of the data and critical review of the manuscript, and all read and approved the final version.

## Authors’ information

Dr. Yamamura is the recipient of a research career award from Founds Choujyu Scientific Academy. Dr. Yamamura is supported by Grants-in-Aid for Scientific Research of Japan. Dr. Mizobata is supported by a Research Grant from Osaka City University.
